# Non-Coding RNA-GATA Axis: Mechanisms and Implications in Cancer Progression and Metastases

**DOI:** 10.3390/cancers18010143

**Published:** 2025-12-31

**Authors:** Aviral Kumar, Uzini Devi Daimary, Mangala Hegde, Mohamed Abbas, Mohammed S. Alqahtani, Hassan Ali Almubarak, Vinay Tergaonkar, Gautam Sethi, Ajaikumar B. Kunnumakkara

**Affiliations:** 1 Cancer Biology Laboratory, Department of Biosciences and Bioengineering, Indian Institute of Technology Guwahati (IITG), Guwahati 781039, Assam, India; 2 Electrical Engineering Department, College of Engineering, King Khalid University, Abha 61421, Saudi Arabia; 3 Radiological Sciences Department, College of Applied Medical Sciences, King Khalid University, Abha 61421, Saudi Arabia; 4 BioImaging Unit, Space Research Centre, Michael Atiyah Building, University of Leicester, Leicester LE1 7RH, UK; 5 Division of Radiology, Department of Medicine, College of Medicine and Surgery, King Khalid University, Abha 61421, Saudi Arabia; 6 Institute of Molecular and Cell Biology (IMCB), Agency for Science, Technology and Research (A*STAR), 61 Biopolis Drive, Singapore 138673, Singapore; 7 Department of Pharmacology and NUS Centre for Cancer Research (N2CR), Yong Loo Lin School of Medicine, National University of Singapore, Singapore 117600, Singapore

**Keywords:** GATA, miRNAs, lncRNAs, circRNAs, EMT, metastasis

## Abstract

GATA transcription factors, defined by their zinc finger DNA-binding domains, are central regulators of tissue development. They modulate gene expression by activating or repressing transcription, thereby coordinating cellular differentiation and cell cycle exit to maintain homeostasis. In progenitor cells, GATA factors promote proliferation, whereas in differentiating cells, they drive maturation and induce cell cycle arrest. Dysregulation of GATA factors has been linked to tumorigenesis and contributes significantly to cancer progression and metastasis. Mutations in GATA factor genes correlate with poor prognosis in multiple cancers, where they influence key oncogenic processes, including sustained proliferative signaling, activation of epithelial–mesenchymal transition, angiogenesis, resistance to cell death, and immune escape. Importantly, their context-dependent roles across tumor types highlight the complexity of their functions in malignancies. Meanwhile, non-coding RNAs have emerged as critical regulators of gene expression, acting as either tumor suppressors or oncogenes by modulating chromatin dynamics, transcription factor activity, and mRNA stability. Despite this, the regulation of GATA transcriptional activity by non-coding RNAs remains largely unexplored. This review highlights the role of GATA factors in regulating EMT and metastasis and focuses on the interplay between non-coding RNAs and GATA transcription factors in cancer progression, proposing a novel regulatory axis with potential implications for biomarker discovery and therapeutic targeting.

## Simple Summary

GATA transcription factors are well known to regulate diverse biological processes of growth, differentiation, development and immunity. Aberrant mutations in these critical regulators result in cancer initiation and progression where they drive different hallmarks of cancer. Non-coding RNAs also regulate gene expression through post transcriptional inactivation or translational block and have been implicated in tumor growth and dissemination. Despite their individual importance, the interplay between GATA transcription factors and non-coding RNAs in cancer remains largely unexplored. This review discusses how these two regulatory systems work together to influence cancer development and progression. Understanding the GATA-non-coding RNA network may reveal new molecular targets and help in designing more effective therapeutic strategies in the future.

## 1. Introduction

**Figure 1:**
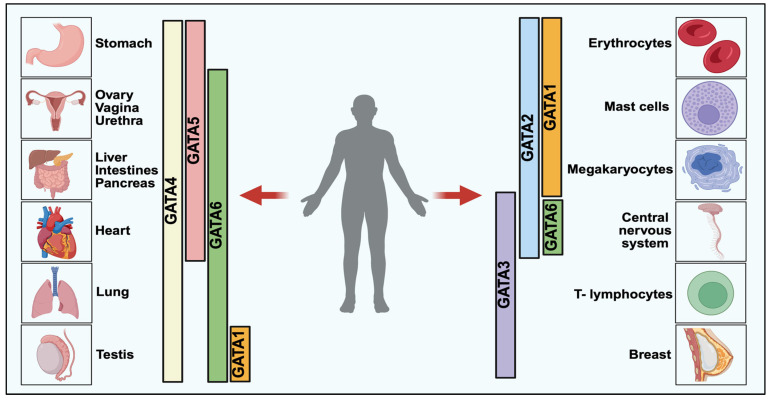
GATA Transcription Factors Expression in Vertebrate Development [5]. GATA transcription factors are categorized into two major classes based on their functional and tissue-specific roles: GATA1/2/3, predominantly involved in hematopoietic lineage regulation, and GATA4/5/6, which play critical roles in endodermal tissue development. Each member exhibits tissue-specific expression, with GATA1–3 largely expressed in blood and immune cells, while GATA4–6 is enriched in organs such as the liver, pancreas, lung, and gut. These factors orchestrate key biological processes including cellular differentiation, lineage commitment, and organogenesis.

The equilibrium of biological processes is maintained by the precise regulation of gene expression, which governs vital life functions. In this complex nexus, at the center, transcription factors play key roles in modulating gene expression involved in differentiation, development, homeostasis, and immunity. Since its discovery in 1988, GATA transcription factors have been implicated to play major roles in development and differentiation [1,2]. As their name suggests, they bind to the A/T GATA A/G elements in the genome and have little conservation in their structural components outside the zinc finger domains [3]. GATA factors exhibit prominent pleiotropic functions in lineage specification from stem/progenitor cells, offering crucial insights into cellular and molecular mechanisms driving cell diversification [1,4]. Accumulating evidence dictates that they activate tissue type-specific genes, which leads to the suppression of alternate genetic programs through feed-forward loops, lineage-specific transcription factors or antagonisms between various regulators of cell fate. These processes result in a cascade of regulatory changes that establish the fate of progenitor cells, thereby enabling the progression of the morphogenetic program [1,5]. GATA transcription factors are evolutionarily conserved across plants, fungi and animals [6]. Vertebrates have six homologs, initially divided into two subfamilies based on function and spatio-temporal expression: GATA (1/2/3), classified as hematopoietic factors, and GATA (4/5/6), considered endodermal factors (Figure 1). However, these factors are broadly expressed across various organs and tissues [7]. Given their importance as developmental regulators, it is unsurprising that many GATA factors have been implicated in multiple human diseases, including cancers [3,5,8,9]. A plethora of studies have delineated the importance of GATA transcription factors in regulating the cellular events leading to oncogenic transformations and metastasis. For instance, GATA3 is crucial for mammary epithelial cell differentiation, and it is lost during luminal breast cancer progression [10]. Recently, immunohistochemistry using GATA3 demonstrated high diagnostic efficacy for malignant breast cancer and helped reduce false negative results in patients [11]. In another study, the GATA4-Notch4-IRG1 network was associated with sporadic colorectal cancer and inflammation and can be utilized as a potential target for obesity and early-onset colorectal cancer (CRC) patients [12]. GATA6 expression could distinguish between the classical and chemoresistant pancreatic ductal adenocarcinoma patients [13]. As GATA1 is a crucial regulator of erythropoiesis and hematopoietic differentiation, mutations in this protein are associated with blood cancers [14,15]. Various other mechanisms also affect cancer progression and understanding the intricate cross talk of transcription factors, signaling molecules is crucial for better therapeutic options in cancer [16,17,18]. Hence, understanding the transcription mediated by GATA factors is essential for elucidating the mechanistic pathways and networks involved in cancer progression, thereby facilitating the development of effective therapeutic strategies.

Non-coding RNAs (ncRNAs), master regulators of gene expression, form intricate networks with nucleic acids and proteins, significantly impacting cellular functions [19]. Although cancer research has largely focused on protein-coding genes, the realization that ~97% of the human genome is non-protein-coding prompted investigations into this “genetic dark matter” in cancer [20]. NcRNAs are often categorized by length: short (19–31 nucleotides), mid (20–200 nucleotides), and long (>200 nucleotides). The most thoroughly investigated in cancer are microRNAs (miRNAs), which are short ncRNAs (22–25 nucleotides in length) [21]. Notably, ncRNAs can influence the survival and fate of cancer cells through various mechanisms, such as altering transcriptional and post-transcriptional processes, modifying chromatin structure, and affecting signal transduction pathways [20,21]. Despite this, the precise roles, and mechanisms of many ncRNAs remain unclear. These molecules form an intricate network of interactions and can function either as oncogenes or tumor suppressors [22,23]. This review describes the functional role of GATA transcription factors in modulating cancer progression and explores their reciprocal regulation with ncRNAs, highlighting a regulatory axis that influences gene expression during cancer progression and metastasis.

## 2. GATA Transcription Factors in Cancer Progression and Metastasis

The GATA transcription family consists of six members, and all are modulators of differentiation and specification of different tissues [1]. GATA factors have two C_2_H_2_ conserved zinc finger domains mediating DNA binding and protein interactions. Among the GATA transcription factors, GATA1, GATA2, and GATA3 have attracted significant attention for their critical roles in embryogenesis, hematopoiesis, and tissue-specific differentiation. GATA1 primarily regulates erythropoiesis and megakaryopoiesis, while GATA2 is essential for hematopoietic stem cell self-renewal and lineage commitment [24,25]. In contrast, GATA3 is crucial for T-cell development and epithelial cell differentiation [10,26]. Meanwhile, GATA4, GATA5, and GATA6 are primarily involved in the differentiation and development of mesodermal and endodermal tissues [5,27]. This includes the induction of embryonic stem cell differentiation, cardiovascular development during embryogenesis, and the regulation of epithelial cell differentiation in adults [5,28].

GATA factors, unlike many other transcription factors, can bind to heterochromatin regions and initiate chromatin remodeling, thereby facilitating the recruitment of additional regulatory complexes to modulate gene expression [1]. They employ several mechanisms to achieve this. First, GATA proteins can function as pioneer factors, opening compact chromatin and enabling the binding of other transcriptional regulators at specific genomic elements [29,30,31]. Second, through chromatin looping, they can bridge distant regulatory regions and promoters, promoting long-range transcriptional interactions [1,32,33,34]. Third, the “GATA switch” mechanism allows one GATA factor to activate gene expression at a target locus, after which it may be replaced by another GATA family member that represses transcription [35,36]. In addition, GATA factors can either activate or repress gene expression by interacting with co-activators such as histone methyltransferases and histone acetyltransferases, or with co-repressors including histone demethylases and histone deacetylases [1]. Finally, GATA factors may also regulate transcription by competing for binding at shared regulatory sites, thereby antagonizing each other’s activity [1].

**Figure 2:**
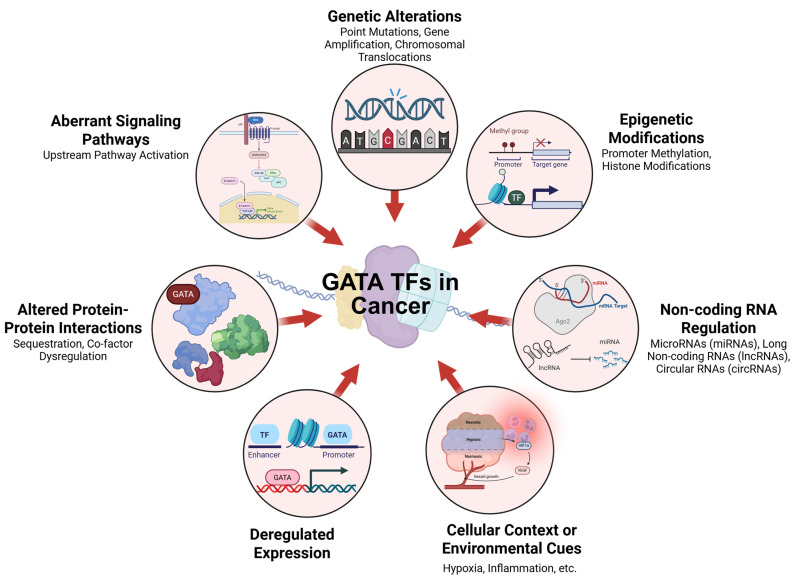
Mechanisms Altering Transcription Factor Activity in Cancer. The diagram summarizes key mechanisms that disrupt transcription factor (TF) activity in cancer. These include genetic changes (mutations, amplifications, translocations), epigenetic modifications, and aberrant signaling pathways. TF function is also influenced by altered protein interactions, deregulated expression, non-coding RNA regulation, and environmental cues, collectively contributing to abnormal gene regulation in tumors.

Increasing evidence implicates aberrant GATA transcription factor expression and function in various cancers, highlighting their dual roles as oncogenes and tumor suppressors (Figure 2) [37,38,39,40]. In leukemia, GATA1 mutations are involved in the pathogenesis of acute megakaryoblastic leukemia (AMKL) and transient myeloproliferative disorder (TMD) in Down syndrome patients [41]. GATA2 dysregulation is associated with myelodysplastic syndromes (MDSs) and acute myeloid leukemia (AML), where it promotes leukemic stem cell self-renewal and impedes myeloid differentiation [42]. GATA2 has been shown to play a pivotal role in promoting metastasis and tumor progression across various cancers. For instance, in pancreatic cancer, GATA2 was found to bind to the promoter regions of Notch3 and transcriptionally activate its expression, thereby promoting the metastasis of pancreatic cancer cells to the liver. Conversely, downregulation of GATA2 markedly reduced the metastatic potential of these cells, highlighting its critical role in pancreatic cancer dissemination [43]. In cervical cancer, high GATA2 expression has been significantly correlated with poor patient prognosis. Functional studies revealed that silencing GATA2 resulted in a pronounced decrease in cancer cell stemness and metastatic ability. Mechanistically, TRIP4 was found to activate GATA2 transcription by binding to its promoter region, forming a TRIP4-GATA2 axis that may represent a potential therapeutic target for limiting metastasis and disease progression [44].

GATA3 has emerged as a context-dependent regulator of metastasis, with both oncogenic and tumor-suppressive roles reported across cancer types. For instance, in breast cancer, GATA3 acts as a tumor suppressor, regulating luminal differentiation and inhibiting epithelial-to-mesenchymal transition (EMT) and metastasis [45,46]. Conversely, in triple-negative breast cancer (TNBC) and estrogen receptor-negative (ER-) tumors, GATA3 expression is often downregulated, correlating with poor prognosis and aggressive disease phenotypes [47,48]. Further, the GATA3-ELK3 axis was shown to be crucial for the metastatic potential of MDA-MB-231 cells, functioning through the modulation of cell–cell adhesion molecules such as claudin, occludin, and ZO-1. Alterations in this axis disrupt epithelial integrity, thereby enhancing invasiveness [49]. Liu et al. (2018) [50] demonstrated that GATA3, in cooperation with its co-factor FOG2, upregulated lysyl hydroxylase (LH) family members, leading to increased migration, invasion, and metastasis in lung cancer. Notably, GATA3 directly activates LH gene transcription, and re-expression of LH2 was sufficient to restore metastatic capability in GATA3-depleted cells, indicating a GATA3-FOG2-LH axis that drives metastatic progression [50]. Conversely, in breast cancer, BRCA1 depletion was shown to induce hypermethylation of the GATA3 promoter, resulting in transcriptional silencing and loss of epithelial characteristics. In GATA3-deficient mouse models, tumors were poorly differentiated and exhibited enhanced EMT and metastatic potential, demonstrating that BRCA1-mediated regulation of GATA3 is essential for maintaining epithelial differentiation and restraining tumor dissemination [46].

Emerging evidence also implicates GATA factors in regulating tumor microenvironment (TME) dynamics. For example, GATA3 influences immune cell infiltration and function within the TME, modulating anti-tumor immunity in breast cancers [3,51]. Additionally, GATA2 governs endothelial cell fate and angiogenesis, fostering tumor growth and metastasis [52]. The multifaceted involvement of GATA transcription factors in cancer pathogenesis positions them as promising therapeutic targets. Targeting GATA1 and GATA2 in leukemia can potentially disrupt leukemic stem cell self-renewal and promote differentiation, offering novel avenues for AML and MDS treatment [3]. Strategies to restore GATA3 expression or function in solid tumors could reprogram tumor cells towards a less aggressive phenotype, enhancing therapeutic responses and patient outcomes [53,54]. Furthermore, the interplay between GATA factors and the TME reveals new opportunities for immunomodulatory therapies. For instance, modulating GATA3 activity in tumor-infiltrating lymphocytes (TILs) could bolster anti-tumor immunity and enhance the efficacy of immune checkpoint inhibitors in GATA3-low cancers [55,56,57]. Additionally, targeting GATA2-mediated angiogenesis could disrupt tumor vascularization and sensitize tumors to anti-angiogenic therapies [52].

Although GATA4, GATA5 and GATA6 are known to be associated with endoderm differentiation, recent studies over few years have demystified their role in cancer progression. For instance, GATA4 with GATA6 regulatory network was responsible for gastric cancer development [58]. Further, KLFF5 interacted with GATA factors and overexpression of these proteins resulted in upregulation of pro-oncogenic transcriptional networks [58]. Further, in another study it was observed that GATA4 played a role in suppressing tumors through non-cell autonomous mechanisms across various models. This tumor-suppressive effect was dependent on cytotoxic CD8 T cells and was partially influenced by the secretion of the chemokine CCL2 [59]. A recent study identified GATA4 as a tumor suppressor that represses MMP9 transcription. Mechanistically, GATA4 interacts with p65 at the NF-κB binding site within the MMP9 promoter, thereby inhibiting its activation. This interaction recruits HDAC1, leading to reduced acetylation of p65 and transcriptional repression. In an in vivo murine breast-cancer model, GATA4 over-expression markedly inhibited metastasis, emphasizing its inhibitory role in tumor invasion [60].

GATA5 functions as a tumor suppressor in prostate cancer by inhibiting its progression through the regulation of PLAGL2 through FAK/AKT/PI3K pathway [61]. Ji et al. (2022) [62] elucidated the tumor-suppressive function of GATA5 in lung adenocarcinoma. Both GATA5 and its downstream target ARHGAP9 were downregulated in tumors, and low ARHGAP9 expression correlated with poor prognosis. GATA5 transcriptionally activated ARHGAP9, and restoration of ARHGAP9 expression significantly inhibited proliferation, migration, and invasion of lung-cancer cells, revealing the GATA5-ARHGAP9 axis as a potential therapeutic target [62]. In one study, methylation of the GATA5 CpG island was identified as a potential biomarker for tumor progression. Moreover, the association between GATA5 hypermethylation and both metastasis and progression-free survival suggests that epigenetic changes in GATA5 contribute to renal cell carcinoma development [63]. Ectopic expression of GATA5 decreased cell proliferation, invasion, migration, and the expression of reprogramming genes such as Nanog, KLF4, c-myc, and β-catenin in hepatocellular carcinoma. Furthermore, GATA5 was shown to interact with β-catenin, preventing its nuclear translocation [64].

It was observed that GATA6 decreased lung cancer cell growth through inhibition of AKT activation which enhanced the expression of p53 and p21 mRNA. This led to stabilization of the p21 protein and promotion of cellular senescence in lung cancer cells [65]. Notably, in one study, it was found that GATA6 expression was high in gastric cancer only when GATA2 CpG was hypermethylated. Moreover, overexpression of GATA2 led to a significant reduction in GATA6 levels, indicating that GATA2 directly suppresses GATA6 expression [66]. GATA6 down-regulation largely due to promoter hypermethylation was strongly associated with lymph-node metastasis in bladder cancer. GATA6 acts as a transcriptional repressor by binding the VEGF-C promoter, and its restoration reduced lymph-node metastasis [67]. In cholangiocarcinoma (CCA), Deng et al. (2020) [68] found that GATA6 expression positively correlated with EMT-related genes such as Vimentin and E-cadherin. GATA6 activated MUC1 by binding to the GATA motifs in its promoter; elevated MUC1 which in turn increased β-catenin expression, driving EMT and invasion [68]. In another study, silencing GATA6 enhanced EMT and lymph-node metastasis in oral cancer. Mechanistically, GATA6 regulated Annexin A10 (ANXA10), which suppressed Vimentin expression, indicating that the GATA6-ANXA10 axis restricts EMT and could serve as an anti-metastatic therapeutic target [69]. Collectively, these studies demonstrate the diverse and context-dependent roles of the GATA transcription-factor family in regulating metastasis and EMT. As observed, GATA transcription factors play a crucial role in tumorigenesis; however, their functions are highly context dependent and vary across cancer types, warranting further investigation. It has been reported that GATA factors can act either as tumor suppressors or oncogenes, depending on a complex interplay of the chromatin environment, cell type, and associated co-factors [70]. For instance, GATA4 and GATA6 exert opposing effects in different cancers, which are influenced by chromatin remodeling and differential co-factor binding [71,72,73]. GATA4 exhibits oncogenic behavior in nasopharyngeal carcinoma, where it promotes proliferation and EMT through regulation of Slug [74]. Furthermore, a genome-wide study reported that GATA factors associate with other transcription factors, such as NR2F2 and FOXA1, to modulate context-specific transcriptional activity, particularly in hormone-driven cancers like breast cancer [75]. Differential chromatin accessibility and co-activator recruitment are key factors contributing to the context-specific functions of GATA factors in tumorigenesis. For instance, one study demonstrated that GATA3 modulates breast cancer progression through chromatin remodeling, achieved via nucleosome eviction, nucleosome sliding, and modulation of local histone marks [76]. In addition, differential expression of ncRNAs can regulate GATA factor expression, thereby further contributing to their context-dependent behavior in cancer [77,78,79]. Understanding these distinct GATA associated molecular pathways may aid in identifying novel biomarkers and therapeutic targets to mitigate cancer invasion and metastatic progression.

Various studies have also potentiated the use of GATA transcription factors as biomarkers in cancer research. For instance, Zhou et al. investigated the prognostic significance of various GATA family members in ovarian cancer. Their analysis revealed that elevated levels of GATA1, GATA2, and GATA4 correlated with improved overall survival, whereas increased expression of GATA3 and GATA6 was linked to poor prognosis. These patterns suggest that GATA factors could serve as prognostic biomarkers in ovarian cancer [80]. Another study used GATA6 expression as a biomarker to distinguish basal-like from classical pancreatic cancer subtypes [13]. Similarly, low GATA6 expression was found to be associated with lymph node metastasis and could be used as a predictive marker for reduced survival in bladder cancer [67]. These studies potentiate the use of GATA factors as diagnostic and prognostic markers for different cancers. Most studies of GATA transcription factors in cancer have been largely correlative, focusing on expression patterns; additional research is needed to fully uncover their roles and significance in cancer development and progression. Elucidating the context-specific functions of GATA factors in cancer biology is essential for developing innovative therapies targeting these regulators.

## 3. Non-Coding RNAs Regulating GATA Transcription Factors

**Figure 3:**
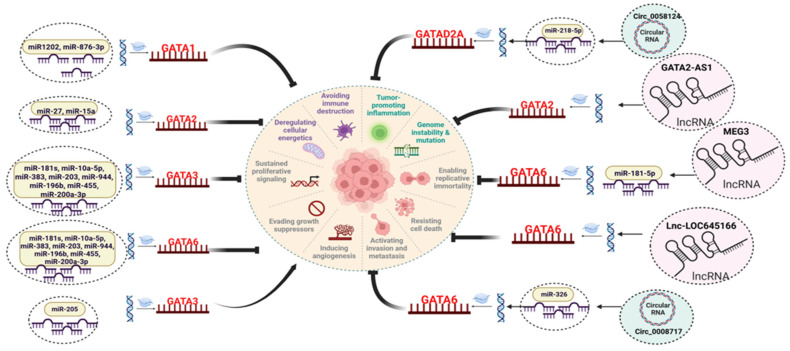
NcRNAs regulating the GATA factors expression in different cancers. Different types of ncRNAs, including microRNAs (miRNAs), long non-coding RNAs (lncRNAs), and circular RNAs (circRNAs), have been identified as key modulators of GATA expression, either directly or indirectly. MiRNAs modulate the expression of GATA factors by binding to its 3′ UTR region of mRNA leading to post-transcriptional inhibition. LncRNAs are known to sequester the miRNAs as competing endogenous RNAs preventing the degradation of RNA. Further, lncRNAs can directly bind to the proteins acting as scaffold for binding of other regulatory proteins. CircRNAs are structurally stable molecules, and they interact with various molecules, such as proteins and miRNAs, to carry out their regulatory functions. This modulation by various ncRNAs significantly influences the various hallmarks of tumorigenesis.

As ncRNAs are well-known crucial modulators of gene expression, it is unsurprising that they regulate GATA factor activity via post-transcriptional inhibition. This section reviews studies demonstrating the roles of various ncRNAs including miRNAs, long non-coding RNAs (lncRNAs), and circular RNAs (circRNAs) in modulating GATA factor expression. Such modulation has a significant impact on key tumorigenic processes like proliferation, apoptosis, invasion, migration, angiogenesis, and metastasis. Given their differential expression in tumors, these ncRNAs can act as tumor suppressors or oncogenes (Table 1) [81]. Here, we discuss the mechanisms by which these diverse ncRNAs influence GATA transcription factor expression, highlighting their critical roles in tumorigenesis (Figure 3).

### 3.1. miRNAs as Modulators of GATA Factors

The revolutionary discovery of miRNAs ushered in a new era in gene regulation, increasing our understanding of the untapped regions in the genome. miRNAs are small, endogenous RNA molecules that are involved in regulating varied biological processes such as division, differentiation, apoptosis, immune response, and homeostasis maintenance [99,100]. miRNAs are regulated by competing endogenous RNAs (ceRNAs) which share similar miRNA recognition elements (MREs) in their sequence [101]. Different types of ceRNAs exist based on their functions; miRNA sponges contain multiple tandem binding sites for the miRNA thereby regulating its activity [102]. Various studies have shown that protein coding RNAs regulate each other by competitive binding with the target miRNAs. Further, other ncRNAs such as lncRNAs and circRNAs also perform the role of ceRNAs by sequestering the miRNA binding sites, thereby regulating their target genes [103]. The deregulation of miRNAs can result from various mechanisms, such as defects in miRNA biogenesis, mutations in miRNA genes, and aberrant epigenetic and transcriptional controls [104]. Numerous studies have elucidated the functional roles of miRNAs in modulating the pathophysiology of various cancers [105,106,107,108]. In the context of cancer, miRNAs are categorized as either oncogenic or tumor-suppressive, depending on their spatiotemporal role [99]. Notably, there have been reports demonstrating that miRNAs can target and regulate GATA transcription factors by binding to the 3′ UTR regions of the target mRNA leading to post transcriptional regulation (Table 2). For instance, Ji J et al. [85] identified distinct miRNA signatures in hepatic stem cell-like hepatocellular carcinoma (HCC), specifically in epithelial cellular adhesion molecule-positive (EpCAM)(+) cells from alpha fetoprotein-positive (AFP)(+) tumors. The study revealed significant overexpression of the miR-181 family in EpCAM(+) AFP(+) clinical samples and HCC cell lines [85]. Notably, the knockdown of miR-181 resulted in the reduced spheroid formation and tumor initiation capability, accompanied by the downregulation of cyclin D1 (*CCND1*) and epithelial cell adhesion molecule (TACSTD1) levels. Furthermore, GATA6 was identified as a direct target of miR-181, suggesting that targeting miR-181 could be a promising strategy to mitigate HCC [85].

miR-196b was found to be highly expressed in non-small cell lung carcinoma (NSCLC) patient samples compared to controls. Using a luciferase reporter assay, GATA6 was identified as a direct target of miR-196b [87]. Overexpression of miR-196b promoted migration and invasion in NSCLC cells, while introduction of GATA6 attenuated these effects. Additionally, miR-196b expression was negatively correlated with GATA6 levels in NSCLC tissues [87]. These findings suggest that miR-196b enhances NSCLC cell migration and invasion by downregulating GATA6, highlighting its potential as a diagnostic marker and therapeutic target for NSCLC. Another study elucidated the functional relationship between the expression of miR-200a-3p and GATA6 and their clinical significance in NSCLC. It was observed that miR-200a-3p was upregulated, and GATA6 was downregulated in the peripheral blood of NSCLC patients, which correlated with poor clinical outcomes and high diagnostic efficiency. Additionally, miR-200a-3p was found to directly target GATA6, suggesting its potential as a diagnostic biomarker for NSCLC [88].

In another study, miR-203 was also found to target GATA6 directly by binding to its 3′ UTR. It was demonstrated that miR-203 overexpression inhibited stemness markers such as CD44, KLF4, and Nanog in HCT-116 and HT-29 cells [112]. Moreover, GATA6 knockdown also exhibited a reduction in stem cell properties in CRC cells. In vivo experiments in nude mice using GATA6 overexpressed HT-29 cells showed higher stem cell subpopulations in GATA6 upregulated cohorts [112]. In a similar study, miR-944 was identified as a tumor suppressor in CRC, with its decreased expression significantly associated with CRC progression [113]. Overexpression of miR-944 reduced the proliferation, invasion, and migration of HCT-116 and SW480 CRC cells, while knockdown of miR-944 enhanced these malignant behaviors. Mechanistically, miR-944 directly targets GATA6, resulting in decreased expression of its downstream effectors, CRT and p-AKT. Furthermore, knockdown of GATA6 reversed the pro-tumor effects induced by miR-944 inhibition, highlighting the critical regulatory role of this pathway in CRC [113]. Poyyakkara A and group investigated the functional effect of integrin β4 (ITGB4) in downstream targets and miRNAs related to EMT. The induction of ITGB4 resulted in the activation of EMT and related signaling pathways such as AKT and β-catenin in Hela cells [89]. As miR-383 was found to be significantly downregulated, it was further ectopically expressed, reversing the high migration potential activated by induction of ITGB4. Moreover, miR-383 targeted GATA6 post-transcriptionally, and the knockdown of GATA6 using shRNA also resulted in the reversal of β-catenin and EMT signaling induced by ectopic expression of ITGB4 [89]. Moreover, similar results were observed in the in ovo model where GATA6 shRNA and miR-383 could reverse the effects of ITGB4 signaling [89].

Recently, in a study, the functional mechanism of GATA6 in ovarian cancer was elucidated, and GATA6 was found to target miR-10a-5p, which were negatively correlated with each other [110]. Lentivirus-mediated upregulation of miR-10a-5p resulted in a reduction in proliferation, colony formation ability, migration, and invasion of SKOV3 and OVCAR3 cells [110]. Moreover, different downstream targets were also found to be decreased in miR-10a-5p overexpressed cells, such as p-AKT, mucin-1(MUC1), and N-cadherin. The in vivo results were consistent with the in vitro results where overexpression of miR-10a-5p led to suppression of tumor growth [110]. As it is well known that hypoxic conditions are a common attribute in retinoblastoma, Xu X et al. investigated the functions of miR-181b in response to hypoxic conditions. It was observed that miR-181b was overexpressed in treatment with hypoxia-induced factor-1α (HIF-1α) in a dose-dependent manner. Moreover, increased expression of miR-181b led to downregulation of GATA6 and PDCD10, with increased capillary tube formation in human umbilical vein endothelial cells (HUVECs) [86]. Hence, targeting this miRNA could be a promising target against this deadly malady. In another study, the expression of insulin-like growth factor binding protein 3 (IGFBP3) was upregulated by GATA1, a transcription factor targeted by miR-876-3p [115]. Notably, miR-876-3p was predominantly enriched in extracellular vesicles (EVs) derived from cancer-associated fibroblasts of patient origin (CAF-P). Administration of CAF-P-derived EVs containing a miR-876-3p antagomir resulted in a reduction in cisplatin resistance compared to the treatment with control miRNA-carrying CAF-P-derived EVs [115].

miR-425-3p was found to target genes such as GATA2, MMP15, IGFBP4 and PDE4B [90]. Originating from exosomes of cancer cells, miR-425-3p inhibited the proliferation and differentiation of preadipocytes, increased lipolysis in adipocytes, and facilitated the browning of white adipocytes. These effects contribute to the loss of adipose tissue observed in cancer cachexia [90]. GATA3 is generally involved in the epithelium development, and alterations in this factor have been reported in various malignancies [10,118]. It was observed that GATA3 expression was increased in the Hut78 T-cell lymphoma cell lines compared to appropriate controls. miR-135a targeted GATA3, and ectopic expression of miR-135a mimics resulted in decreased GATA3 and thymocyte selection associated high mobility group box (TOX) expression [84]. In cervical cancer, miR-205 was found to be upregulated, while GATA3 was downregulated, suggesting a negative correlation. miR-205 directly targeted GATA3, and its overexpression enhanced cell viability, migration, and tube formation in cervical cancer cells [111]. Conversely, downregulation of miR-205 reversed these effects. Importantly, restoring GATA3 expression counteracted the oncogenic effects of miR-205, indicating a functional miR-205/GATA3 regulatory axis that may influence cervical cancer progression [111]. Various other studies have also potentiated the role of miRNAs in regulating the GATA transcriptional activity in different cancers [77,78]. Taken together, further research must be conducted to delve into miRNAs’ transcriptional regulation of GATA factors, which could provide insights for developing novel therapeutic strategies to combat this disease.

### 3.2. LncRNAs as Modulators of GATA Factors

LncRNAs, typically around 200 nucleotides in length, modulate gene expression by acting as decoys, scaffolds, enhancers, and guides, thereby regulating DNA, RNA, and protein expression [119,120]. Moreover, lncRNAs can function as ceRNAs, sequestering miRNAs by sharing similar miRNA response elements, which affects the expression of miRNAs and their target genes [120]. Moreover, lncRNAs influence gene transcription by facilitating or preventing the formation of transcriptional loops and by either attracting or hindering regulatory factors. In one study, it was observed that lncRNA ZNF503-AS1 interacted with GATA6 to modulate the expression of calcium channel protein SLC8A1 in bladder cancer [121]. Numerous studies have linked aberrant lncRNA expression to tumor progression [122,123,124]. Some studies have also demonstrated that certain lncRNAs sequester miRNAs that target GATA factors or by directly interacting with other regulatory proteins, thus modulating the activity of these transcription factors (Table 2).

#### 3.2.1. LncRNAs Act as ceRNAs to Regulate GATA Factors

Various studies have potentiated the role of lncRNAs to function as ceRNAs thereby modulating GATA factors gene expression. For instance, one study found that GATA6 and lncRNA maternally expressed gene 3 (MEG3) were upregulated in prostate cancer clinical samples. Treatment with niraparib increased MEG3 expression with inhibition of proliferation, migration, and invasion of PC3 cell lines [79]. Further, GATA6 was found to be directly targeted by miR-181-5p, and overexpression of this miRNA reversed the inhibitory effects of MEG3 upregulation on cancer cell proliferation. Further, the knockdown of MEG3 reversed the suppression of niraparib-mediated tumor growth in murine models [79]. Another study revealed that lncRNA keratin 16 pseudogene 6 (lncKRT16P6) was upregulated in tongue squamous cell carcinoma (TSCC), and knockdown of lncKRT16P6 was associated with decreased proliferation, migration, and invasion of TSCC cells [95]. Further, miR-3180 was found to be the direct target of lncKRT16P6, and this lncRNA acted as ceRNA to regulate the GATA zinc finger domain containing 2A expression [95]. GATA6-AS1, a long non-coding RNA, is downregulated in gastric cancer and functions as a tumor suppressor; wherein it inhibited gastric cancer cell proliferation and migration both in vitro and in vivo. Mechanistically, GATA6-AS1 acts as a ceRNA for miR-543, leading to increased PTEN expression and suppression of the AKT signaling pathway [94]. In chronic myelogenous leukemia (CML), the long non-coding RNA SNHG5 was upregulated and promoted leukemic cell proliferation. Knockdown of SNHG5 in K562 cells inhibited proliferation, induced differentiation as evidenced by upregulation of GATA1, CD14, CD42b, CD11b, and β-globin. Mechanistically, SNHG5 suppression increases DR4 expression by reducing its promoter methylation, highlighting a potential therapeutic strategy for CML [116].

#### 3.2.2. LncRNAs Bind with the DNA Elements to Regulate GATA Factors

LncRNAs also have the ability to interact with the DNA elements that regulate GATA gene expression. For example, GATA2 and its counterpart lncRNA GATA2 antisense RNA 1 (GATA2-AS1) were found to be upregulated in colon cancer. Downregulation of both lncRNA and GATA factors resulted in decreased proliferation, EMT, and invasion with increased apoptosis [96]. It was observed that lncRNA GATA2-AS1 helped in the recruitment of DDX3X which stabilized GATA2 mRNA. In a feedback loop mechanism, GATA2 binds in the promoter regions of GATA2-AS1, thereby enhancing its transcription [96]. In one study, SNHG16-L was found to suppress GATA3 transcription in ovarian cancer cells by binding to CCAAT/enhancer-binding protein B (C/EBPβ). This interaction reduced cancer cell migration and invasion while enhancing sensitivity to paclitaxel treatment [117]. Hence, lncRNAs acting as ceRNAs modulate the expression of GATA factors, which could provide valuable insights into the etiology of various cancers.

### 3.3. CircRNAs as Modulators of GATA Factors

CircRNAs are single-stranded RNA molecules characterized by their closed-loop structure, which is produced through exon-skipping events or back-splicing of precursor mRNAs in eukaryotic cells [125]. It is formed between the 5′ upstream splice site and the 3′ downstream splice site transcribed by RNA polymerase II [126]. These small single-stranded RNA molecules are stable in cytosol and function by interacting with other RNAs and proteins [127]. Recent studies have delineated the importance of circRNAs in tumor progression [128,129,130]. Although circRNAs are known to modulate gene expression, there are limited reports on their regulation of GATA factors’ transcriptional activity (Table 2). For instance, Yang L and the group explored the mechanism of circ_0008717 in breast cancer pathogenesis. It was found that circ_0008717 and GATA6 were upregulated in breast cancer tissues and cell lines. Circ_0008717 was observed to act as a sponge for miR-326, whose target was GATA6 [98]. Knockdown of circ_0008717 attenuated different hallmarks of cancer both in vitro and in vivo. Ectopic expression of miR-326 in rescue experiments reversed the anticancer effects of circ_0008717 knockdown [98]. Hence, circ_0008717 could act as a potential target for breast cancer treatment. Although circRNA research is in its infancy, further research is required to delve into the intricate regulatory network of GATA factors and circRNAs in cancer.

## 4. GATA Transcription Factors Regulating Non-Coding RNA Transcription

**Figure 4:**
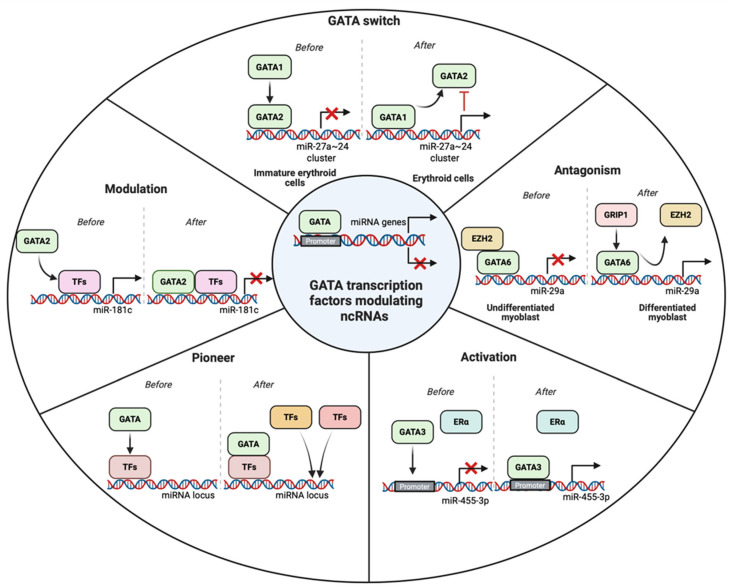
GATA transcription factors modulating the transcription of ncRNAs using various approaches such as activation, antagonism, modulation, pioneer, and GATA switch. Activation depicts the binding of GATA factors to the promoter of the target ncRNAs. Antagonism refers to the displacement of the repressor transcription factors (TFs) leading to transcriptional activity. Modulation happens when the GATA factors bind to TFs already bonded in the DNA to repress the activity of transcription. When GATA factors bind with the regulatory TFs to call other TFs and adaptor proteins in the promoter elements, it is a pioneer activity. GATA switch refers to the displacement of GATA factors to regulate the activity of ncRNAs transcription.

As GATA transcription factors are well-known regulators of diverse biological and physiological processes, it is not unexpected that they can also regulate the biogenesis of ncRNAs by binding at the regulatory elements in their promoters or repressing the activity of other related proteins involved in ncRNA transcription (Table 3 and Figure 4).

### 4.1. GATA Factors as Promoters of ncRNA Expression

GATA transcription factors can promote the ncRNA biogenesis by binding to the DNA elements and recruitment of various other co-activators or by GATA switch. For example, Song et al. studied the causative factors of rhabdomyosarcoma genesis and the dysregulated myogenic differentiation. They discovered that in undifferentiated C2C12 myoblasts, GATA4 recruits the polycomb protein EZH2 to suppress miR-29a expression. During differentiation, GRIP-1 binds to GATA4, leading to the eviction of EZH2 and subsequent upregulation of miR-29a [131]. A comparable regulatory pattern is seen in rhabdomyosarcoma cells, where EZH2 remains associated with the miR-29a promoter, maintaining transcriptional repression. However, upon re-differentiation toward skeletal muscle lineage, EZH2 is displaced, allowing miR-29a reactivation [131]. These findings highlight GATA4’s role as a regulatory switch in the control of miR-29a and underscore its tumor-suppressive potential in rhabdomyosarcoma, offering a promising avenue for therapeutic intervention [131]. A plethora of studies have implicated GATA factors as master regulators of complex transcriptional networks in erythropoiesis [24,135,136]. A study exploring the interplay between GATA transcription factors and miRNAs in CML revealed that GATA1 and GATA2 jointly regulate the expression of miR-24 and miR-27a, two microRNAs integral to erythroid lineage commitment [83]. In early progenitor cells, GATA2 suppresses these miRNAs by binding their shared promoter region. As differentiation progresses, GATA1 replaces GATA2 at this site, promoting transcriptional activation [83]. Interestingly, once expressed, miR-24 and miR-27a inhibit GATA2 expression at the post-transcriptional level, thereby reinforcing the dominance of GATA1 and establishing a self-reinforcing regulatory loop conducive to erythroid maturation [83]. GATA3 is crucial for the development of mammary epithelial cells, and alterations in this factor are well-known in breast cancer [10,137]. In one study, GATA3 was found to activate the expression of miR-455-3p by repressing the EMT. ZEB1 was found to be a direct target of miR-455-3p [91]. It was observed that ZEB1 recruited the NuRD complex near the promoter regions of miR-455-3p to suppress the GATA3-mediated activation of miRNA. Upregulation of miR-455-3p inhibited EMT by targeting components of the TGF-β signaling pathway [91].

In another study, miR-125a-5p was shown to play a key role in modulating the conversion of regulatory T (Treg) cells under IL-6-rich inflammatory conditions. Under steady-state conditions, miR-125a-5p expression was low in Treg cells; however, its expression could be upregulated by GATA3, leading to the suppression of IL-6R and STAT3 expression [133]. This regulatory mechanism highlights the involvement of the GATA3/miR-125a-5p/IL-6R-STAT3 axis in maintaining FOXP3 stability and Treg cell identity [133]. In a study, it was found that a potential SNP rs4938723 (T > C) increased the risk of hepatocellular carcinoma. Further, this SNP was reported near the pri-miR-34b/c promoter regions, which could affect the GATA binding site. Furthermore, SNP rs4938723 had a significant association with alcohol intake and HCC risk [134]. GATA1 was found to transcriptionally activate the lncRNA LINC01503 in carboplatin-resistant ovarian cancer cells. LINC01503 was upregulated in these cells and acted as a sponge for miR-766-5p, which directly targeted PD-L1 [97]. Overexpression of miR-766-5p enhanced carboplatin sensitivity by downregulating PD-L1, highlighting the role of the GATA1/LINC01503/miR-766-5p/PD-L1 axis in chemoresistance [97].

### 4.2. GATA Factors as Repressors of ncRNA Expression

Previous reports have also shown that GATA factors can repress the ncRNA biogenesis. For instance, in GATA2-deficient hematopoietic cells, miR-181c expression was found to be upregulated, leading to the downregulation of MCL1, a key pro-survival factor. Functional assays in HL-60 and other leukemia cell lines confirmed that elevated miR-181c promotes apoptosis by directly targeting MCL1. Moreover, experiments demonstrated that GATA2 represses the transcription of miR-181c, and its knockdown results in heightened miR-181c levels and reduced MCL1 expression. These findings suggest that GATA2 deficiency may contribute to increased cell death and cytopenia through a miR-181c/MCL1 regulatory axis [132]. Yang Y and the group demonstrated Jagged2 as a promoter of metastasis through the upregulation of GATA factors, which in turn suppress the activity of the miR-200 family. These miRNAs are found to target transcriptional repressors that induce EMT. In this regard, miR-200 decreased GATA3 expression, thereby reversing EMT and inhibiting metastasis. Moreover, GATA3 was found to bind to the promoter regions of miR-200b and suppress its transcriptional activation [109]. The miR-497~195 cluster was found to be progressively downregulated with increasing meningioma grade. This downregulation was mediated by GATA4, a transcription factor upregulated in malignant meningiomas, which suppressed the miRNA cluster and promoted cell viability. This was associated with increased expression of cyclin D1, a key cell cycle regulator [92]. Although GATA factors can also modulate the transcriptional status of ncRNAs, further studies are imperative to build a regulatory interconnected network with feedback loops, which could be employed to better understand the pathophysiology of cancers.

## 5. Discussion and Perspective

The GATA transcription factor family is composed of six proteins (GATA1–6) that play roles in numerous physiological and pathological activities. The embryonic lethality in single GATA knockout mice demonstrated the critical role of GATA factors in development [5]. Additionally, mutations in GATA genes are linked to various human disorders, including cancer, renal insufficiency syndrome, hypoparathyroidism, congenital heart diseases, and sensorineural deafness [1,3,7,8]. A plethora of studies have implicated GATA transcription factors and their alterations in various cancers. For instance, notably, the absence of GATA3 expression in ER+ breast cancers is correlated with resistance to hormonal therapy and poor prognosis [138]. Although GATA3 inhibits tumor formation in the breast, it appears to be involved in the tumorigenesis of lymphoid precursor cells [139]. In mice, overexpression of GATA3 driven by the human CD2 locus control region results in the development of CD8+ CD4+ double-positive T cell lymphomas. GATA2 has also been shown to be essential for oncogenic KRAS-driven lung tumorigenesis. Furthermore, inhibiting GATA2-associated signaling pathways in mice with KRAS mutant NSCLC leads to tumor regression [140]. While significant research over the past decade has unraveled aspects of the GATA factor enigma, the application of GATA factors in cancer clinical management remains in its early stages. A comprehensive understanding of their regulatory networks and associated proteins is essential for elucidating their role in tumorigenesis.

Despite the highly conserved DNA binding domains across different GATA factors and their recognition of similar GATA motifs, numerous reports suggest that these factors can only partially compensate for each other functionally and activate specific transcriptional programs in distinct cellular contexts. This raises a crucial question: How do GATA factors interact with one another to confer specificity in transcriptional regulation? This indicates that the differences in the regulatory activity of GATA factors are attributable to elements beyond their DNA binding domains and core DNA motifs. However, various studies have revealed that residues inside and around the zinc-finger domains may explain the GATA factor-specific binding preferences [141,142]. For example, it has been proposed that residues in the less conserved C-terminal region (around the second zinc finger) influence lineage specificity, distinguishing endodermal from hematopoietic functions [35]. GATA factors play a crucial role in the development and differentiation of various cell types by activating cell-specific genes and repressing those associated with alternative lineages. The distinction between the activating and repressing activities of GATA factors often arises from their physical interactions and cooperation with other transcription factors, regulatory co-factors, and chromatin modifiers. Herein, another interesting question comes to mind: Is GATA factor regulation spatiotemporal? If so, how do these factors bind to specific regions in the DNA and regulate transcription? Additionally, the GATA switch, a mechanism for altering transcriptional programs during hematopoietic differentiation, is well studied, and documented, and similar GATA switches may occur in other cell types, which warrants further investigation.

Recent studies have elucidated the role of GATA transcription factors in maintaining tumor heterogeneity and shaping the immune microenvironment, with important implications for therapy response and prognosis. For instance, GATA6 was shown to distinguish classical from basal-like molecular subtypes in pancreatic cancer. Tumors with high GATA6 expression exhibited reduced infiltration of immunosuppressive regulatory T cells and M2 macrophages, alongside increased infiltration of activated T cells and antigen-presenting cells, correlating with improved patient survival [143]. In another study, both GATA3 and GATA6 were found to be upregulated in pancreatic cancer, with GATA3 expression closely associated with immune cell infiltration signatures, highlighting its potential role in modulating the tumor microenvironment [144]. Moreover, GATA4 has been reported to regulate a stress-induced secretory program that recruits cytotoxic CD8^+^ T cells, and its loss resulted in diminished lymphocyte infiltration and reduced tumor growth in multiple model systems [59]. These findings suggest that GATA factors not only reflect heterogeneity among tumor cells (e.g., subtype, differentiation state) but also actively drive differences in immunogenicity and immune infiltration. Nevertheless, mechanistic insights into how ncRNAs regulate GATA factors in this context, and how the ncRNA-GATA axis contributes to heterogeneity in antigen expression or immune evasion, remain largely unexplored.

With the advent of high throughput and next-generation technologies, we have deepened our understanding of the functional regulation of ncRNAs in development and disease. Unlike GATA factors, ncRNAs primarily function through post-transcriptional inhibition or translational repression, thereby modulating gene expression. There has been a lot of research undergoing to functionally map and characterize the “dark side of the genome”; however, in certain instances, elucidating the function of non-coding transcripts outside their physiological context proves challenging due to their limited conservation across species. This lack of conservation complicates the translation of in vivo findings to human applications. Additionally, ncRNAs are inherently more difficult to study compared to coding genes. Various studies have implicated ncRNAs to be involved in the initiation and progression of various human disorders, including cancer. In cancer, ncRNAs are classified based on their expression pattern in the tumor, either as oncogenes or tumor suppressors. Understanding the expression of ncRNAs has helped the researchers to distinguish them as potential biomarkers or use them to develop novel RNA based therapeutics. As expected from such pivotal regulators, ncRNAs can influence the expression of other key regulatory proteins, including GATA factors. In turn, there have been few reports where GATA transcription factors modulate the expression of ncRNAs acting as molecular switches and completing the feedback regulatory loops. Despite extensive research on the functional roles of various ncRNAs in tumorigenesis, relatively few studies have investigated their involvement in modulating the activity of GATA transcription factors. In this regard, one very important question arises: to what extent do ncRNAs directly regulate GATA factor activity and can reciprocal GATA-ncRNA feedback loops serve as molecular switches in cancer progression? Looking ahead, several promising research avenues should be explored to deepen our understanding of the intricate regulatory crosstalk between ncRNAs and GATA transcription factors in cancer and their potential roles of the ncRNA-GATA axis in tumor heterogeneity and immunotherapy. One such promising approach is the development of ncRNA-based therapeutics designed to target GATA-regulated gene networks. RNA-based drugs, some of which are already in clinical trials for chronic diseases, highlight the therapeutic relevance of this strategy. Currently, substantial effort has been made in the field of RNA therapeutics mostly using antisense oligonucleotides or small interfering RNAs for different diseases including cancer [145,146]. The diverse functional repertoire of using RNA based therapeutic approach stems down to its broad targeting, diverse mechanisms and disease relevance. An ideal RNA-based therapeutic requires thorough assessment of its immunogenic potential, strategic chemical modifications to improve stability and biological performance, and delivery approaches optimized for effective biodistribution and intracellular release [145]. Moreover, it must precisely and efficiently interact with its target molecule and be administered at an appropriate dosage to produce the intended therapeutic effect [145]. Effective delivery of ncRNAs to the target site is challenging owing to the negatively charged and hydrophilic nature of oligonucleotides, which prevents cellular uptake [147]. Furthermore, their short half-lives lead to rapid degradation [147]. Recent advancements have resulted in improved cellular uptake and more efficient pharmacokinetics of ncRNAs. First- and second-generation chemical modifications have resulted in increased stability, improved protein interactions, and enhanced resistance to nuclease-mediated degradation [148]. Although third-generation oligonucleotides are highly stable, they exhibit limitations in cellular uptake and often require higher doses [145,149]. Various delivery approaches, such as nanoparticles, have been developed, including polymer- and lipid-based vectors, conjugated vectors, and metal-based nanoparticles [150,151].

Another valuable approach lies in utilizing chromatin accessibility assays like ATAC-Seq to delineate the context-dependent binding patterns of GATA factors across different cancer types. When integrated with methods such as ChIP-Seq or CUT&RUN, these profiles can reveal how chromatin architecture and accessibility potentially influenced by ncRNAs guide GATA factor recruitment to specific genomic loci including direct GATA interactions with ncRNAs. Furthermore, high-throughput CRISPR-based screening platforms offer powerful tools to functionally characterize ncRNAs involved in regulating GATA expression and activity. These screens can uncover novel components of the ncRNA-GATA axis, including enhancer RNAs and regulatory lncRNAs, paving the way for the discovery of new molecular targets. Collectively, these integrative approaches promise not only to advance our fundamental understanding of ncRNA-mediated regulation of GATA transcription factors but also to open new avenues for precision oncology by targeting the molecular determinants of cellular identity and plasticity in tumors.

## 6. Conclusions

GATA transcription factors have been extensively studied for their pleiotropic function in development, differentiation, and immunity. They work as transcriptional regulators by recruiting and interacting with other co-activators or repressors to modulate gene expression. Accumulating evidence suggests that the aberrant functions of GATA factors contribute to tumorigenesis. These factors operate in a context-dependent manner, acting as both oncogenic and tumour suppressor agents based on the tumour cell type and its microenvironment. NcRNAs, on the other hand, regulate gene expression through post-transcriptional mechanisms and play a major role in tumour progression and metastasis. In this review, we examine the reciprocal regulatory interactions between ncRNAs and GATA transcription factors, highlighting a feedback network that regulates tumorigenesis. Furthermore, we discuss the pleiotropic functions of these RNA molecules at multiple levels of transcriptional regulation and consider them as potential targets for novel diagnostic and therapeutic strategies. Although individual research articles have reported ncRNAs targeting GATA factors or GATA factors influencing ncRNA biogenesis, a comprehensive compilation and conceptual framework unifying these findings has not yet been presented. Our review aims to fill this gap by offering the first focused overview of this regulatory axis and its significance in cancer biology. Although this review compiles the current knowledge on the ncRNA-GATA axis in cancer progression and metastasis, several limitations still exist. Most available studies have predominantly focused on well-characterized ncRNAs such as miRNAs, lncRNAs, and circRNAs, whereas emerging ncRNA classes including piRNAs, snoRNAs, and others remain largely unexamined. Future research exploring these lesser-studied ncRNAs would significantly broaden our understanding of this regulatory axis in cancer biology. Further, most of the studies are performed in cell-based assay and lack in vivo validation which is crucial for development of novel therapeutics. Moreover, integrating multi-omics approaches, including transcriptomic, proteomic, epigenomic, and single-cell analyses, will be essential to obtaining a holistic, systems-level representation of the underlying mechanisms.

Given the regulatory capacity of miRNAs, lncRNAs, and circRNAs in influencing GATA factor expression and function, synthetic RNA molecules such as antisense oligo-nucleotides and RNA interference agents could be tailored to counteract GATA-related transcriptional abnormalities in cancer. Furthermore, recent progress in targeted delivery technologies, including lipid-based nanoparticles and exosome-derived carriers, along with chemical modifications that enhance RNA stability and bioavailability, offers significant promise for improving the precision and effectiveness of such treatments. Several studies have shown that GATA factors can function as pioneer factors or participate in GATA-switch mechanisms that influence ncRNA biogenesis. Future research should also explore whether a direct GATA-ncRNA axis mediates epigenetic regulation in cancer, either through chromatin remodeling or through the recruitment of co-factors involved in epigenetic modification or whether there are direct interactions present between GATA and ncRNAs. Understanding these mechanisms would open new avenues for developing tailored therapeutic strategies for different cancer types. Finally, well-designed, random, multi-centered clinical trials are needed to validate preclinical findings and advance the development of novel therapeutic modalities for cancer.

## Figures and Tables

**Table 1:** Various non-coding RNAs regulating GATA transcription factors and their expression in different model systems.

Non-Coding RNA	Cancer	In Silico/In Vitro/In Vivo/Clinical	Model	Expression	References
**MicroRNAs**					
miR-15a	Osteosarcoma	Clinical	Tumor tissues	Down	[82]
		In vitro	HOS, SaOS-2, MG-63, U2-OS		
miR-23a~27a~24-2 cluster	Erythroleukemia	In vitro	Hemin treated K562s	Up	[83]
miR-135a	Cutaneous T-cell lymphoma	In vitro	Hut78	Down	[84]
miR-181	Hepatocellular carcinoma	Clinical	Hepatic Stem Cell-like tumor tissues, and Fetal liver	Up	[85]
			Mature Hepatocyte-like tumor tissues	Down	
		In vitro	Hep3B cells	Up	
			EpCAM(+) HuH1 or HuH7 cells	Up	
miR-181b	Retinoblastoma	In vitro	HXO-RB44	Up	[86]
miR-196b	Non-small cell lung carcinoma	Clinical	Tumor tissue samples	Up	[87]
miR-200a-3p	Non-small cell lung carcinoma	In silico	LUAD datasets from TCGA	Up	[88]
		Clinical	Peripheral blood from patients		
miR-383	Cervical Cancer	In vitro	ITGB4CD overexpression in HeLa	Down	[89]
Exosomal miR-425-3p	Cachexia-inducing tumour	In vitro	A549, H1299, and AGS	Up	[90]
miR-455-3p	Breast cancer	In silico	GEO datasets	Down	[91]
miR-497~195 cluster	Malignant meningioma	Clinical	Tumor tissues	Down	[92]
		In vitro	KT21-MG1-Luc5D		
miR-720	Breast cancer	In vitro	Tumor associated macrophages and monocyte-derived macrophages	Down	[78]
			M2-polarized THP-1 cells		
miR-1202	Transient abnormal myelopoiesis	Clinical	Residual peripheral blood samples or bone marrow	Down	[93]
**Long non-coding RNAs**					
MEG3	Prostate Cancer	Clinical	Tumor tissues	Down	[79]
GATA6-AS1	Gastric cancer	In silico	STAD datasets from TCGA	Down	[94]
		In vitro	HGC-27, MKN-7, MKN-45	Down	
lncKRT16P6	Tongue squamous cell carcinoma	Clinical	Tumor tissues	Up	[95]
		In vitro	CAL-27, SCC-9	Up	
GATA2-AS1	Colorectal cancer	In silico	COAD datasets from TCGA/NCBI datasets	Up	[96]
		In vitro	SW480, HCT-116, DLD-1, HT-29, SW620	Up	
LINC01503	Ovarian cancer	In vitro	OVCAR-3/CBP and CAOV-3/CBP	Up	[97]
**CircRNAs**					
Circ_0008717	Breast cancer	Clinical	Tumor tissues	Up	[98]
		In vitro	MCF-7, MDA-MB-231		

EpCAM(+): Epithelial cellular adhesion molecule-positive; LUAD: Lung adenocarcinoma; ITGB4CD: Integrin subunit beta 4 gene cytoplasmic domain; GEO: Gene Expression Omnibus; STAD: Stomach adenocarcinoma; COAD: Colon Adenocarcinoma; CBP: Carboplatin.

**Table 2:** Various non-coding RNAs and their regulation of GATA transcription factors in cancer.

Non-Coding RNA	Cancer	Target	In Vitro/In Vivo	Model	Mechanistic Effect	References
**MicroRNAs**						
miR-181s ^b,^*	Hepatic cancer	GATA6	In vitro	HuH1	↓ Spheroid formation, ↑ UGT2B7, ↑ CYP3A4, ↓ cyclin D1, ↓ TACSTD1, ↑ CDX2, ↑ NLK	[85]
miR-206 ^a^	Breast cancer	GATA3	In vitro	MCF-7	↓ Proliferation, ↓ BrdU-labeled cells, ↑ Apoptosis, ↓ cyclin D1, ↓ Bcl-2, ↑ cleaved PARP	[77]
miR-200b ^a^	Non-small cell lung carcinoma	ZEB1	In vitro	H322	Indirect targeting GATA3	[109]
miR-181b ^b^	Retinoblastoma	-	In vitro	HXO-RB44	↑ Capillary tube formation,↑ PDCD10, ↑ GATA6	[86]
miR-10a-5p ^a^	Ovarian cancer	GATA6	In vitro	SKOV3 and OVCAR3	↓ Proliferation, ↓ Colony formation, ↓ N-cadherin, ↑ E-cadherin, ↓ p-AKT(S473), ↓ MUC1	[110]
			In vivo	SKOV3/pLV-miR-10a xenograft	↓ Tumor volume, ↓ Ki67, ↓ N-cadherin, ↑ E-cadherin, ↓ p-AKT(S473)	
miR-383 ^a,c^	Cervical cancer	GATA6	In vitro	ITGB4CD overexpression in Hela	↓ Migration, ↓ Cyclin D1, ↓ SNAI1, ↓ FN1, ↑ E-cadherin, ↓ FSP1	[89]
			In ovo	ITGB4CD with miR-383 co-expressed in Hela xenograft	↓ Tumor Weight, ↓ FSP1, ↓ Vimentin, ↓ α-SMA	
miR-15a ^a^	Osteosarcoma	GATA2	In vitro	MG-63	↓ Colony formation, ↓ Migration, ↓ Invasion, ↓ Bcl-2, ↑ BAX, ↓ CDK2, ↓ CDK4, ↓ cyclin D, ↓ cyclin E, ↑ p53	[82]
			In vivo	MG-63 xenograft	↓ Tumor volume, ↓ Tumor weight	
miR-205 ^a^	Cervical cancer	GATA3	In vitro	-	↑ Cell viability, ↑ Migration, ↑ Tube formation	[111]
miR-203 ^a^	Colorectal cancer	GATA6	In vitro	HCT-116, HT-29	↓ Colony formation, ↓ Migration, ↓ Sphere formation, ↓ CD44, ↓ KLF4, ↓ Oct4, ↓ Nanog	[112]
miR-135a ^a^	Cutaneous T-cell lymphoma	GATA3	In vitro	Hut78	↓ Proliferation, ↓ TOX	[84]
miR-944 ^a^	Colorectal cancer	GATA6	In vitro	HCT116, SW480	↓ Proliferation, ↓ Colony formation, ↓ Migration, ↓ Invasion, ↓ p-AKT, ↓ CRT, ↓ GATA6	[113]
miR-196b ^a^	Lung cancer	GATA6	In vitro	A549, H226	↑ Migration, ↑ Invasion	[87]
miR-27a ^a^	Myelogenous leukemia	GATA2	In vitro	K562	↓ GATA2	[114]
miR-720 ^a^	Breast cancer	GATA3	In vitro	M2-polarized THP-1	↓ CD163, ↓ IL-10, ↓ CCL17, ↑ M2 polarization	[78]
miR-200a-3p	Non-small cell lung carcinoma	GATA6	Clinical	Peripheral blood from patients	Poor prognosis and overall survival	[88]
miR1202 ^a^	Myeloid leukaemia	GATA1	In vitro	K562	↓ GATA-1_s_ isoform, ↓ Proliferation, ↑ Apoptosis, ↑ BAX, ↓ Bcl-xL	[93]
Exosomal miR-876-3p ^a^ from cancer associated fibroblast	Oral squamous cell carcinoma	GATA1	In vitro	FaDu, UMSCC1	↓ GATA1, ↓ IGFBP3, ↑ Cisplatin resistance, ↑ Cisplatin treated organoid size	[115]
Exosomal miR-876-3p ^b^ from cancer associated fibroblast			In vivo	FaDu spheroids + cancer associated fibroblast xenograft	↓ Tumor size, ↑ GATA1 ↑ IGFBP3, ↑ Cisplatin sensitivity	
Exosomal miR-425-3p	Cachexia-induced tumour	GATA2	In vitro	Human preadipocytes-visceral (HPA-v)	↓ Proliferation, ↓ Differentiation, ↓ ADPN, ↓ aP2, ↓ IGFBP4, ↓ MMP15, ↑ UCP1, ↑ PKA, ↑ LC3-II/LC3-I ratio, ↑ cAMP, ↑ Glycerol release, ↑ p-CREB, ↑ p-HSL, ↑ p-PLIN1, ↓ PDE4B	[90]
**Long non-coding RNAs**						
MEG3 ^a^	Prostate cancer	miR-181-5p/GATA6	In vitro	PC3	↓ Proliferation, ↓ Migration, ↓ Invasion, ↑ ICAM-1, ↑ CD44, ↓ E-cadherin	[79]
MEG3 ^b^ + Niraparib			In vivo	PC3 xenograft	↑ Tumor weight, ↑ Ki67	
GATA6-AS1 ^a^	Gastric cancer	miR-543	In vitro	MKN-45, AGS	↓ Proliferation, ↓ Colony formation, ↓ Migration, ↓ Invasion, ↑ PTEN, ↓ p-AKT	[94]
			In vivo	GATA6-AS1-overexpressed MKN-45	↓ Tumor volume, ↓ Tumor weight, ↑ PTEN	
lncKRT16P6 ^b^	Tongue squamous cell carcinoma	miR-3180/GATAD2A	In vitro	CAL-27, SCC-9	↓ Proliferation, ↓ Colony formation, ↓ Migration, ↓ Invasion, ↑ Apoptosis, ↑ G0/G1 populations, ↓ CDK2, ↓ cyclin E, ↓ cyclin A, ↓ cyclin D1	[95]
			In vivo	CAL-27 xenograft	↓ Tumor volume, ↓ Tumor weight, ↓ Ki67	
SNHG5 ^b^	Chronic myelogenous leukemia	-	In vitro	K562	↓ Proliferation, ↑ Apoptosis, ↓ CDK2, ↓ cyclin E1, ↑ p27, ↑ CD42b, ↑ CD11b, ↑ CD14, ↑ GATA-1, ↑ β-globin	[116]
GATA2-AS1 ^b^	Colorectal cancer	DDX3X	In vitro	SW480, HCT-116	↓ Proliferation, ↓ Colony formation, ↓ EdU^+^ cells, ↑ Apoptosis, ↓ GATA2, ↑ E-cadherin, ↓ N-cadherin, ↓ Vimentin, ↓ Nanog, ↓ Oct4	[96]
			In vivo	sh-GATA2-AS1 transfected HCT-116 or SW480 xenograft	↓ Tumor volume, ↓ Tumor weight, ↓ Ki67, ↓ PCNA, ↑ E-cadherin, ↓ N-cadherin, ↓ Nanog, ↓ Oct4	
SNHG16-L ^a^	Ovarian cancer	GATA3	In vitro	SKOV3	↓ Migration, ↓ Invasion, ↑ E-cadherin, ↓ N-cadherin, ↓Vimentin	[117]
**Circular RNAs**						
Circ_0008717 ^b^	Breast cancer	miR-326/GATA6	In vitro	MCF-7, MDA-MB-231	↓ Proliferation, ↓ Colony formation, ↓ Migration, ↓ Invasion, ↑ E-cadherin, ↓ N-cadherin, ↓ Vimentin	[98]
			In vivo	MDA-MB-231 xenograft	↓ Tumor volume, ↓ Tumor weight, ↑ E-cadherin, ↓ N-cadherin, ↓ Vimentin	

^a^: Overexpression; ^b^: Knockdown; ^c^: co-expression; ^*^: family. ↓: Downregulation and ↑: Upregulation. ITGB4CD: Integrin subunit beta 4 gene cytoplasmic domain; UGT2B7: Uridine diphosphate-glucuronosyltransferase 2B7; CYP3A4: cytochrome P450 family 3 subfamily A member 4; CDX2: Caudal type homeobox 2; NLK: Nemo like kinase; AKT: Protein Kinase B; MUC1: Mucin 1; SNAI1: Snail family transcriptional repressor 1; FN1: Fibronectin 1; FSP1: Ferroptosis suppressor protein 1; α-SMA: α-smooth muscle actin; MEG3: Maternally expressed 3 gene; ICAM-1: Intercellular adhesion molecule-1; GATA6-AS1: GATA6 antisense RNA 1; PTEN: Phosphatase and tensin homolog; PARP: Poly(ADP-ribose) polymerase; CDK: Cyclin-dependent kinase; IGFBP3: Insulin-like growth factor binding protein; TOX: Thymocyte selection-associated HMG-box gene; KLF4: Kruppel-like factor 4; BAX: Bcl-2-associated protein x

**Table 3:** GATA transcription factors regulating the expression of non-coding RNAs.

GATA Transcription Family	Cancer	Model	Non-Coding RNAs	Effect	References
GATA4	Rhabdomyosarcoma	C2C12 cells	Promote miR-29a transcription	↓ Myogenic differentiation, ↑ EZH2 mediated regulation	[131]
GATA2 ^b^	Leukemia	HL-60, Ly-8, and SP53 cells	Repress miR-181c transcription	↑ p53	[132]
GATA2 ^b^ + miR181c ^a^				↑ Apoptosis, ↓ Proliferation, ↓ MCL1	
GATA1 ^a^	Erythroleukemia	Hemin treated K562s cells	Promote transcription of miR-23a∼27a∼24-2 cluster	↓ GATA2	[83]
GATA3 ^b^	Breast cancer	MCF7 cells	Promote miR-455-3p transcription	↓ α-catenin, ↓ E-cadherin, ↑ N-cadherin, ↑ fibronectin	[91]
GATA4 ^b^	Malignant meningioma	Ben-Men-1 cells	Repress miR-497~195 cluster	↑ Proliferation, ↑ Bcl-2, ↑ cyclin D1, ↑ p-RB	[92]
GATA1 ^a^	Ovarian cancer	OVCAR-3/CBP and CAOV-3/CBP resistant cell	Promote LINC01503 expression	↓ miR-766-5p	[97]
GATA3 ^a^	Acute lymphocyte leukemia	Jurkat cells	Promote miR-125a-5p	↓ Treg sensitivity	[133]
GATA	Hepatocellular carcinoma	Patients	Regulate pri-miR-34b/c expression	Increased risk due to presence of SNP rs4938723 in miR promoter	[134]
GATA3 ^b^	Non-small cell lung carcinoma	H322 cells	miR-200b	↓ Invasion, ↓ TGF-β induced EMT	[109]

^a^: Overexpression; ^b^: Knockdown. ↓: Downregulation and ↑: Upregulation.

## Abbreviations

The following abbreviations are used in this manuscript:

AML	Acute myeloid leukemia
CAF-P	Cancer-associated fibroblasts of patient origin
CCND1	Cyclin D1
CeRNAs	Competing endogenous RNAs
CircRNAs	Circular RNAs
CML	Chronic myelogenous leukemia
CRC	Colorectal cancer
EMT	Epithelial-to-mesenchymal transition
EpCAM(+)	Epithelial cellular adhesion molecule-positive
ER−	Estrogen receptor-negative
EVs	Extracellular vesicles
GATA2-AS1	GATA2 antisense RNA 1
GATA6-AS1	GATA binding protein 6 antisense RNA 1
HCC	Hepatocellular carcinoma
HIF-1α	Hypoxia induced factor-1α
HUVECs	Human umbilical vein endothelial cells
IGFBP3	Insulin-like growth factor binding protein 3
ITGB4	Integrin β4
LncRNAs	Long non-coding RNAs
MDS	Myelodysplastic syndrome
MEG3	Maternally expressed gene 3
miRNAs	MicroRNAs
MUC1	Mucin 1
ncRNAs	Non-coding RNAs
NSCLC	Non-small cell lung carcinoma
PTC	Papillary thyroid cancer
SNHG5	Small nucleolar RNA host gene 5
TACSTD1	Epithelial cell adhesion molecule
TILs	Tumor-infiltrating lymphocytes
TMD	Transient myeloproliferative disorder
TME	Tumor microenvironment
TNBC	Triple-negative breast cancer
TOX	Thymocyte selection associated high mobility group box
TSCC	Tongue squamous cell carcinoma
